# Leukotriene B_4_ Receptors Are Necessary for the Stimulation of NLRP3 Inflammasome and IL-1β Synthesis in Neutrophil-Dominant Asthmatic Airway Inflammation

**DOI:** 10.3390/biomedicines9050535

**Published:** 2021-05-11

**Authors:** Dong-Wook Kwak, Donghwan Park, Jae-Hong Kim

**Affiliations:** 1 Department of Biotechnology, School of Life Sciences and Biotechnology, Korea University, Seoul 02841, Korea; diaak1992@gmail.com (D.-W.K.); pictogram@korea.ac.kr (D.P.); 2 Division of Life Sciences, College of Life Sciences, Korea University, Seoul 02841, Korea

**Keywords:** neutrophil, airway inflammation, leukotriene B4 receptors, NLRP3 inflammasome, IL-1β

## Abstract

The stimulation of the NOD-, LRR- and pyrin domain-containing protein 3 (NLRP3) inflammasome and IL-1β synthesis are associated with chronic respiratory diseases such as neutrophil-dominant severe asthma. Leukotriene B_4_ (LTB_4_) is a principal chemoattractant molecule for neutrophil recruitment, and its receptors BLT1 and BLT2 have been suggested to contribute to neutrophil-dominant asthmatic airway inflammation. However, the relationship between BLT1/2 and NLRP3 in neutrophil-dominant asthmatic airway inflammation has not been previously studied. In the present study, we investigated whether BLT1/2 play any roles in stimulating the NLRP3 inflammasome and IL-1βsynthesis. The blockade of BLT1 or BLT2 clearly suppressed the stimulation of the NLRP3 inflammasome and IL-1β synthesis in house dust mite (HDM)/lipopolysaccharide (LPS)-induced neutrophilic airway inflammation. The enzymes 5-lipoxygenase and 12-lipoxygenase, which catalyze the synthesis of BLT1/2 ligands [LTB_4_, 12(*S*)-hydroxyeicosatetraenoic acid (12(*S*)-HETE), and 12-hydroxyheptadecatreinoic acid (12-HHT)], were also critically associated with the stimulation of NLRP3 and IL-1β synthesis. Together, our results suggest that the 5-/12-LOX-BLT1/2-linked cascade are necessary for the simulation of the NLRP3 inflammasome and IL-1β synthesis, thus contributing to HDM/LPS-induced neutrophil-dominant airway inflammation.

## 1. Introduction

Asthma is an airway inflammatory disease consisting of various phenotypes that are driven by different pathways. Severe asthma is more strongly associated with Th1/Th17 responses and neutrophilic airway inflammation [1,2,3,4,5,6]. Approximately 5–20% of asthma patients respond poorly to corticosteroids even at high doses, and no effective treatments are currently available for severe, steroid-resistant asthma [3,7,8]. Recent studies have reported that NLRP3-dependent IL-1β is associated with severe neutrophilic asthmatic progression and pathological exacerbation, and the levels of IL-1β in sputum were elevated with the severity of the disease [9,10]. However, the detailed signaling pathway for the synthesis of the NLRP3 inflammasome and IL-1β in neutrophil-dominant asthmatic airway inflammation remains to be determined.

Leukotriene B_4_ (LTB_4_), a product of 5-LOX, is a potent chemoattractant for neutrophils and a key mediator in inflammation [11,12,13]. BLT1, a high-affinity receptor of LTB_4_, is exclusively expressed on the surface of leukocytes. On the other hand, BLT2 has low affinity for LTB_4_, and it is expressed in various tissues, including the spleen, lung, and liver. BLT2 has been shown to interact with various arachidonic acid metabolites, such as 12(*S*)-hydroxyeicosatetraenoic acid (12(*S*)-HETE) or 12-hydroxyheptadecatreinoic acid (12-HHT), in addition to LTB_4_. A previous study demonstrated that LTB_4_ is present in higher concentrations in sputum of severe asthmatics with a relatively large amount of neutrophil influx compared with the sputum of mild asthmatics [14]. Similarly, 12(*S*)-HETE, a 12-LOX product, has been shown to be highly elevated in the serum of the neutrophilic airway inflammation group compared with the healthy control group [15]. In addition, we previously demonstrated that the LTB_4_ receptors BLT1/2 contributed to neutrophilic airway inflammation, especially house dust mite (HDM)/lipopolysaccharide (LPS)-induced neutrophilic airway inflammation and asthmatic exacerbation [6]. However, it remains to be determined whether BLT1/2 are associated with NLRP3 stimulation and IL-1β synthesis in neutrophilic airway inflammation.

In the present study, we observed that BLT1/2 play critical roles in stimulating the NLRP3 inflammasome and further found that BLT1/2 are necessary for NLRP3-dependent IL-1β synthesis. Thus, our studies suggest that the ‘5-/12-LOX-BLT1/2-NLRP3-IL-1β’ signaling cascade potentially contributes to NLRP3 inflammasome stimulation and IL-1β synthesis in HDM/LPS-driven neutrophilic airway inflammation. This finding may provide a novel perspective for the development of new therapeutic targets for neutrophilic severe asthma.

## 2. Materials and Methods

### 2.1. Reagents

Recombinant mouse IL-1β was obtained from R&D Systems (Minneapolis, MN, USA). MCC950 (NLRP3 inhibitor, cat no. inh-mcc) was obtained from Invitrogen (San Diego, CA, USA). Lipopolysaccharide (LPS, Escherichia coli serotype O55:B5), dexamethasone (cat no. D4902), and dimethyl sulfoxide (DMSO) were purchased from Sigma-Aldrich (St. Louis, MO, USA). MK886 (5-LOX inhibitor, cat no. 475889) was obtained from Calbiochem (La Jolla, CA, USA). Ac-YVAD-cmk (caspase-1 inhibitor, cat no. ALX-260-028), U75302 (BLT1 antagonist, BML-RA102-0100) and baicalein (12-LOX inhibitor, BML-EI106-0050) were obtained from Enzo Life Sciences (Farmingdale, NY, USA). LY255283 (BLT2 antagonist, cat no. 70715) was purchased from Cayman Chemical (Ann Arbor, MI, USA). Antibodies against NLRP3 and caspase-1 were purchased from AdipoGen Life Sciences (San Diego, CA, USA).

### 2.2. Model for HDM-Driven Neutrophil-Dominant Airway Inflammation

**Figure 1:**
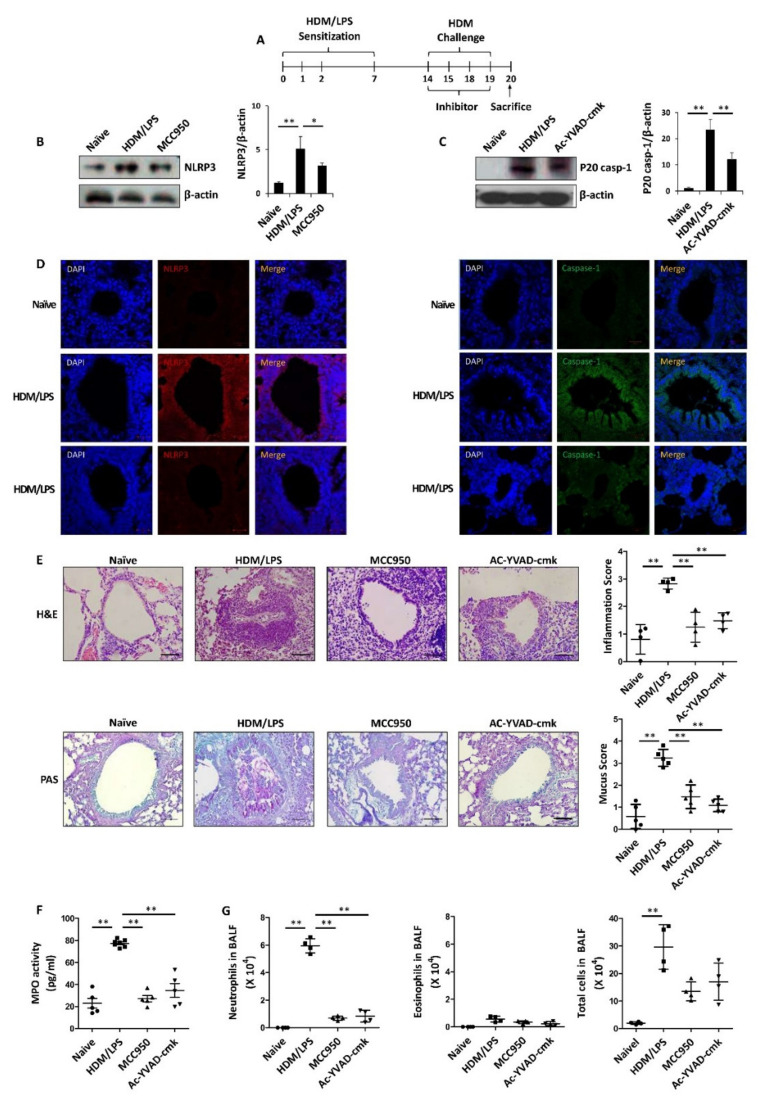
HDM/LPS-driven neutrophilic airway inflammation is dependent on NLRP3 stimulation. For the experiment, we administered the control (DMSO), MCC950 (10 mg/kg) or Ac-YVAD-cmk (1 mg/kg) via i.p. injection 1 h before every challenge (*n* = 4–7 per group). Naïve designates control without any additions. (**A**) Scheme of the HDM/LPS-driven neutrophilic airway inflammation model. Airway inflammation was induced by the immunization with LPS (10 μg)/HDM (100 μg) in 25 μL of saline and the challenge with HDM (50 μg) in 25 μL of saline. (**B**,**C**) The mouse lungs were homogenized and proteins were isolated to assess the levels of NLRP3 or caspase-1 by Western blotting. Data are representative of three independent experiments with similar results. (**D**) Immunofluorescence staining were done in lung tissues for detecting NLRP3 (red, CYTM3) or caspase-1 (green, FITC). Nuclei were stained with DAPI (blue; 400×). The images are representative of three independent experiments with similar results. (**E**) H&E and PAS staining of the lungs. Perivascular and peribronchial lung inflammation as well as mucus secretion were determined and scored (400×). Data are shown as the mean ± SD (*n* = 4–5 per group). (**F**) MPO levels were analyzed in lung lysates using ELISA. (**G**) The numbers of neutrophils and eosinophils as well as total immune cells were obtained in BALF using Cytospin and then stained with Diff-Quik. Data are shown as the mean ± SD (*n* = 4 per group). All experiments were performed in triplicate. * *p* < 0.05, ** *p* < 0.01 versus each control group.

Female BALB/c mice (8 to 10 weeks old) were purchased from Young Bio (Seongnam, Korea). HDM extract was obtained from Greer Laboratories (Lenoir, NC, USA). The endotoxin level present in 100 µg HDM was 329.15 EU. HDM was administered intranasally, with the mice lightly anesthetized with 5% isoflurane before immunization. The mice were intranasally introduced with 10 μg of LPS + 100 μg of HDM on days 0, 1, 2, and 7 and then challenged with 50 μg of HDM on days 14, 15, 18, and 19. The mice were sacrificed on day 20 (Figure 1A). For inhibition experiments, MCC950 (HDM/LPS + 5 mg/kg of MCC950), Ac-YVAD-cmk (1 mg/kg), U75302 (50 μg/kg), LY255283 (10 mg/kg), baicalein (75 mg/kg), MK886 (5 mg/kg) or vehicle control (DMSO; 80 µL DMSO + 20 µL saline) was injected by intraperitoneal (i.p.) administration 1 h before each HDM challenge. For the IL-1β inhibition experiment, either 5 mg/kg control IgG1 or anti-IL-1β antibody in 0.1% methylcellulose (100 µL) was treated to the mice through i.p. injection 1 h before each challenge. The mouse room was controlled at a temperature of 22 ± 1 °C and a humidity of 50 ± 10% with a 12 h light/dark cycle. Wire-lidded food hoppers within the cages were filled to capacity with rodent chow, and the mice had access to water bottles. All experimental animals used for this study were treated according to ARRIVE guidelines approved by the Institutional Animal Care and Use Committee of Korea University (KU-IACUC) and the experimental protocols were approved by KU-IACUC (Approval no. KU-IACUC-2019-0056; Approval date. 21 May 2020).

### 2.3. Preparation of Lung Tissue Lysates and Immunoblot Analysis

Lung tissue lysates were prepared as described previously [6]. The lysate samples were heated at 95 °C for 5 min and subjected to SDS-PAGE. The separated proteins were electrophoretically transferred to a polyvinylidene difluoride membrane for 45 min at 110 V. The membrane was incubated for 1 h with TBS-T containing 5% nonfat dried milk at room temperature (RT) and for 1 h with primary antibodies at a 1:1000 dilution (1:500 for NLRP3, caspase-1, ASC, BLT1 and BLT2 or 1:5000 for β-actin) in TBS-T. The membrane was then incubated for 1 h at RT with HRP-conjugated secondary antibodies before the detection of immune complexes with an enhanced chemiluminescence kit (Amersham Biosciences, Little Chalfont, UK). Proteins were quantified using ImageJ software (NIH, Bethesda, MD, USA)

### 2.4. Measurement of LTB_4_, IL-1β and Myeloperoxidase (MPO)

The levels of LTB_4_, IL-1β and MPO were measured in the lung tissue lysates or serum using an ELISA kit (Abcam, Cambridge, UK) for IL-1β and MPO (Enzo Life Sciences, Farmingdale, NY, USA) for LTB_4_ according to the manufacturer’s instructions.

### 2.5. Bronchoalveolar Lavage (BAL) Cell Counting

BAL cells were collected from bronchoalveolar lavage fluid (BALF) by centrifugation (1000× g for 3 min) and washed with phosphate-buffered saline (PBS). Next, the BAL cells were centrifuged at 500 rpm by cytocentrifuge (CytoSpin, Hanil Science, Gimpo, Korea) before staining. After that, BAL cells were fixed on glass slides and stained with a Diff-Quik kit (Sysmex, Kobe, Japan).

### 2.6. Histological Analysis of Lung Tissues Including Immunofluorescence (IF) Staining

Lung tissues were harvested and fixed in 10% formaldehyde for 10 days and then embedded in paraffin. Lung sections (4.5 μm thick) were next mounted onto Superfrost Plus glass slides (Fisher Scientific, Pittsburgh, PA, USA), deparaffinized and then stained with hematoxylin and eosin (H&E) or periodic acid-Schiff (PAS). All images were prepared using a BX51 microscope (Olympus, Tokyo, Japan) equipped with a DP71 digital camera (Olympus, Tokyo, Japan). For evaluating the degree of peribronchial and perivascular lung inflammation, a subjective scale from 0 to 3 was used. Grade 0 was designated as “no detectable inflammation”, grade 1 was defined as occasional cuffing with inflammatory cells, grade 2 was defined as most bronchi or vessels were detected with a thin (1 to 5 cells thick) layer of inflammatory cells, and grade 3 was designated when most bronchi or vessels were detected with a thick (more than 5 cells thick) layer of inflammatory cells. For quantification of goblet cells in the airway mucus, we used a five-point grading system: 0 < 0.5% PAS positive cells; 1 < 25%; 2, 25–50%; 3, 50–75%; and 4 >75%. The images were acquired using a BX51 microscope (Olympus, Tokyo, Japan) equipped with a DP71 digital camera (Olympus, Tokyo, Japan) [16]. To perform IF staining, lung sections (4.0 μm thick) were precisely mounted onto SuperfrostTM Plus slides (Fisher Scientific, Pittsburgh, PA, USA) and then were deparaffinized, rehydrated, and blocked with buffer (PBS containing 1% BSA) for 1 h at RT. The mouse tissues were then incubated at 4 °C with antibodies against NLRP3 and caspase-1. After washing three times in PBS, the tissue slides were incubated for 1 h at RT with CyTM3-conjugated anti-rabbit IgG (Jackson ImmunoResearch Inc., West Grove, PA, USA), PE-conjugated or FITC-conjugated anti-rabbit IgG (Fisher Scientific, Pittsburgh, PA, USA) diluted in PBS containing 1% BSA. After washing, the slides were incubated with DAPI (Sigma-Aldrich, St. Louis, MO, USA). The slides were washed in PBS, mounted, and observed under a confocal laser scanning microscope (LSM 700, Carl Zeiss, Oberkochen, Germany).

### 2.7. Statistical Analysis

Statistical analyses of the results were performed with one-way ANOVA followed by Tukey’s post hoc test. SPSS software 25 (IBM SPSS Statistics for Windows, version 21.0; IBM Corp., Armonk, NY, USA) and GraphPad Prism 5.0 (GraphPad Software, Inc., San Diego, CA) were used for the statistical analysis. Data are presented as the mean ± standard deviation (SD) from three independent experiments. *p*-values < 0.05 indicated statistical significance.

## 3. Results

### 3.1. Stimulation of the NLRP3 Inflammasome Is Necessary for HDM/LPS-Induced Neutrophilic Airway Inflammation

We first examined whether the stimulation of the NLRP3 inflammasome is associated with HDM/LPS-induced neutrophilic airway inflammation. To do this, we used inhibitors of NLRP3 or caspase-1 in an HDM/LPS-induced mouse model (Figure 1A). The NLRP3 inhibitor MCC950 (5 mg/kg) or the caspase-1-specific inhibitor Ac-YVAD-cmk (1 mg/kg) was intraperitoneally injected 1 h before each challenge. With inhibitor treatment, the levels of NLRP3 and caspase-1 were markedly reduced (Figure 1B,C). By IF staining analysis, NLRP3 and p20 caspase-1 expression were also shown to be markedly reduced by these inhibitors (Figure 1D). Under these experimental conditions, airway inflammation was significantly reduced by MCC950 and Ac-YVAD-cmk, as determined by histological analysis and quantitative analysis of the inflammation and mucus scores (Figure 1E). In addition, the activity of the key enzyme of neutrophil activation, MPO, was substantially reduced by MCC950 and Ac-YVAD-cmk (Figure 1F). Next, we found that the numbers of neutrophils in BALF were significantly decreased by MCC950 and Ac-YVAD-cmk (Figure 1G). Together, these results suggested that the stimulation of the NLRP3 inflammasome is necessary for HDM/LPS-induced neutrophilic airway inflammation.

### 3.2. IL-1β Is Produced via NLRP3 Stimulation and Is Critical for the HDM/LPS-Induced Neutrophilic Airway Inflammation

**Figure 2:**
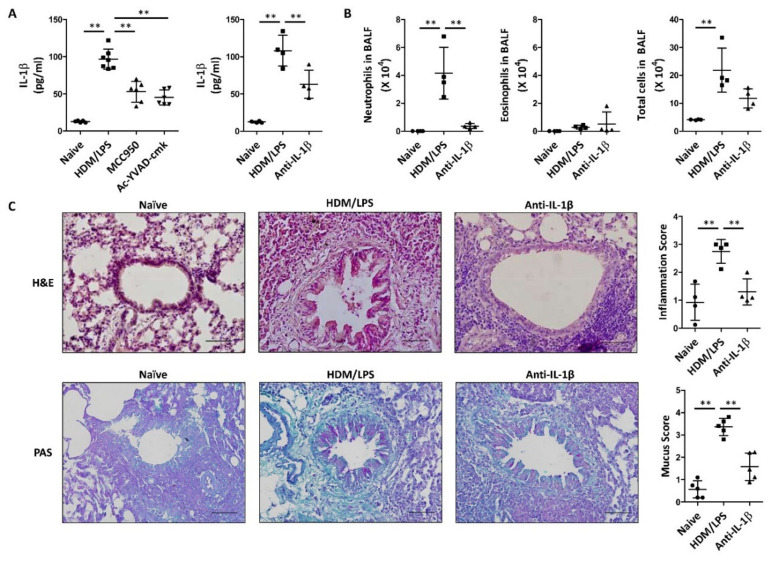
IL-1β is produced via NLRP3 stimulation and is critical for the HDM/LPS-induced neutrophilic airway inflammation. To analyze the effect of IL-1β inhibition, mice were received with anti-IL-1β antibody or control IgG1 (5 mg/kg) via i.p. injection 1 h before each challenge. Naïve designates control without any additions. (**A**) Levels of IL-1β were analyzed in lung lysates using ELISA. Data are shown as the mean ± SD (*n* = 4–7 per group). (**B**) The numbers of neutrophils and eosinophils as well as total immune cells were obtained in BALF using Cytospin and then stained with Diff-Quik. Data are shown as the mean ± SD (*n* = 4 per group). (**C**) H&E and PAS staining were performed with the lungs that were excised and fixed. Perivascular and peribronchial lung inflammation were determined and scored (400×). Data are shown as the mean ± SD (*n* = 4–5 per group). All experiments were performed in triplicate. ** *p* < 0.01 versus each control group.

Next, we analyzed whether the NLRP3-dependent synthesis of IL-1β was associated with HDM/LPS-induced neutrophil-dominant airway inflammation. To do this, an anti-IL-1β neutralizing antibody (5 mg/kg) was administered intraperitoneally 1 h before each challenge. Anti-IL-1β apparently reduced the levels of IL-1β (Figure 2A). Under these experimental conditions, the numbers of neutrophils in BALF were clearly suppressed by an anti-IL-1β neutralizing antibody (Figure 2B). Airway inflammation was also suppressed by the anti-IL-1β neutralizing anti-body, as determined by histology and inflammation and mucus scores (Figure 2C). Thus, these results suggest that NLRP3 inflammasome-dependent IL-1β synthesis is critical for HDM/LPS-induced neutrophilic airway inflammation.

### 3.3. LTB_4_ Receptors Are Necessary for the Stimulation of the NLRP3 Inflammasome and IL-1β Synthesis in HDM/LPS-Induced Neutrophilic Airway Inflammation

**Figure 3:**
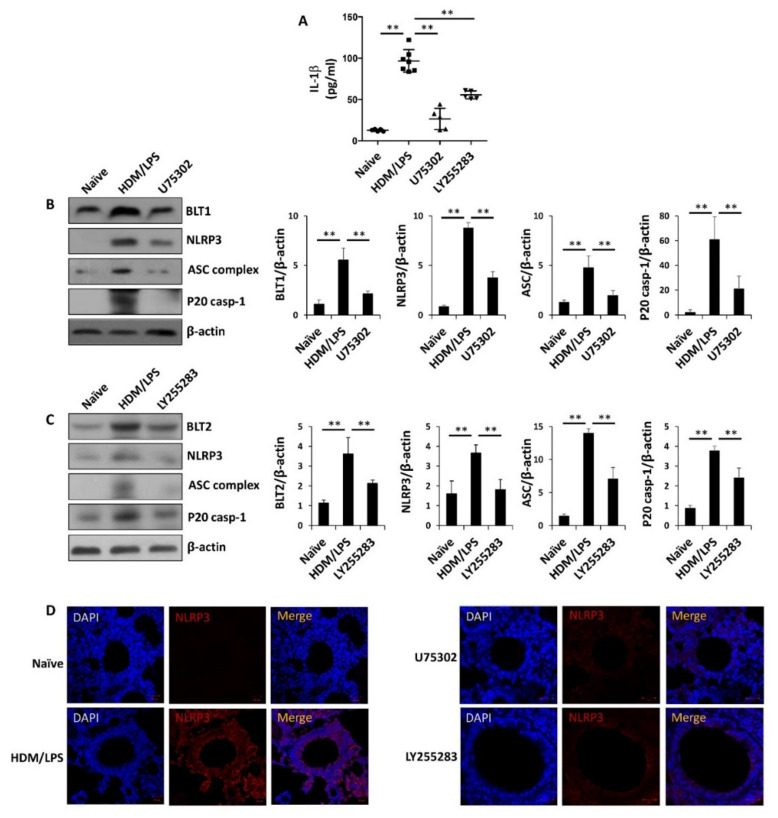

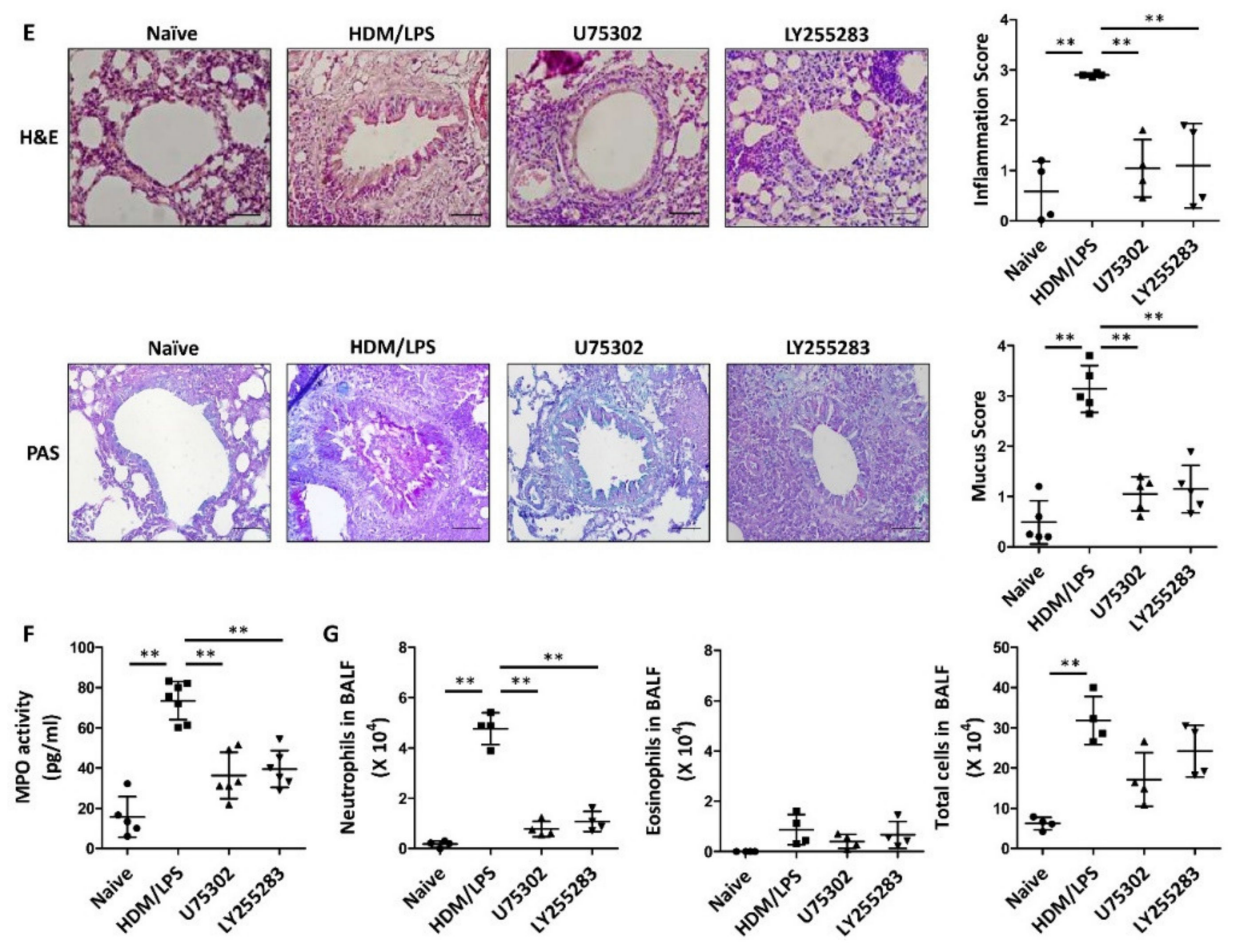
LTB_4_ receptors are necessary for NLRP3 stimulation and IL-1β production in HDM/LPS-induced neutrophilic airway inflammation. The control DMSO, U75302 (50 μg/kg) or LY255283 (10 mg/kg) were administered via i.p. injection 1 h before every challenge. Naïve designates control without any additions. (**A**) Levels of IL-1β in lung lysates were analyzed using ELISA. Data are shown as the mean ± SD (*n* = 57 per group). (**B**,**C**) The mouse lungs were homogenized, protein was isolated, and the levels of BLT1/2, NLRP3, caspase-1, and ASC complex were assessed by Western blotting. Data are representative of three independent experiments with similar results. (**D**) Immunofluorescence staining for NLRP3 (red, CY^TM^3) in lung tissues. Nuclei were stained with DAPI (blue) (400×). The images are representative of three independent experiments with similar results. (**E**) The lungs were excised, fixed and stained with H&E and PAS. Peribronchial and perivascular lung inflammation and goblet cells were measured and scored (400×). Data are shown as the mean ± SD (*n* = 4–5 per group). (**F**) Levels of MPO in lung lysates were analyzed using ELISA. Data are shown as the mean ± SD (*n* = 5–7 per group). (**G**) Neutrophils, eosinophils, and total immune cells in BALF were obtained using Cytospin and stained with Diff-Quik. Data are shown as the mean ± SD (*n* = 4 per group). All experiments were performed in triplicate. ** *p* < 0.01 versus each control group.

To determine the roles of the LTB_4_ receptors BLT1 and BLT2 in the stimulation of the NLRP3 inflammasome and IL-1β synthesis in the HDM/LPS-driven neutrophilic mouse model, we tested the effect of BLT1 or BLT2 inhibition. The BLT1 antagonist U75302 (50 μg/kg) and BLT2 antagonist LY255283 (10 mg/kg) were injected intraperitoneally 1 h before the challenge as previously described (Figure 1A). In response to these inhibitors, the levels of IL-1β were significantly suppressed (Figure 3A). The levels of NLRP3, ASC complex, and caspase-1 were also markedly suppressed by U75302 and LY255283 (Figure 3B,C). By IF staining analysis, NLRP3 expression was also reduced by U75302 and LY255283 (Figure 3D). In addition, the levels of airway inflammation and MPO activity were significantly reduced by U75302 and LY255283, as determined by histological analysis and the quantitative analysis of the inflammation and mucus score (Figure 3E,F). Furthermore, neutrophil numbers in BALF were significantly reduced by U75302 and LY255283 (Figure 3G). Taken together, these results suggest that BLT1/2 are necessary for the stimulation of the NLRP3 inflammasome and IL-1β synthesis in HDM-induced neutrophilic airway inflammation.

### 3.4. 5-/12-Lipoxygenase Are also Necessary for the Stimulation of the NLRP3 Inflammasome and IL-1β Production in HDM/LPS-Driven Neutrophilic Airway Inflammation

**Figure 4:**
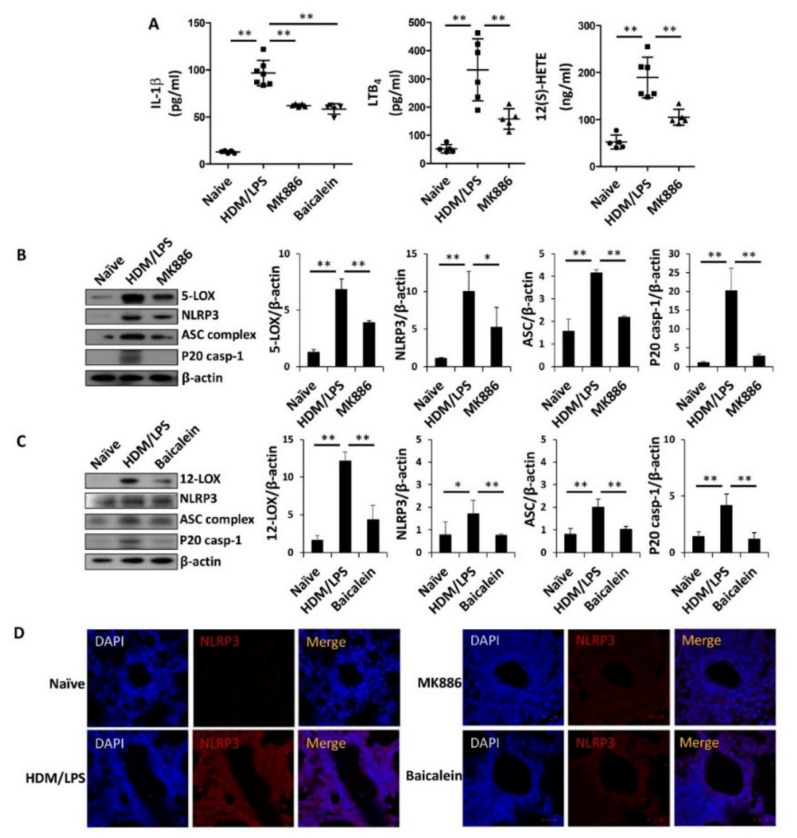

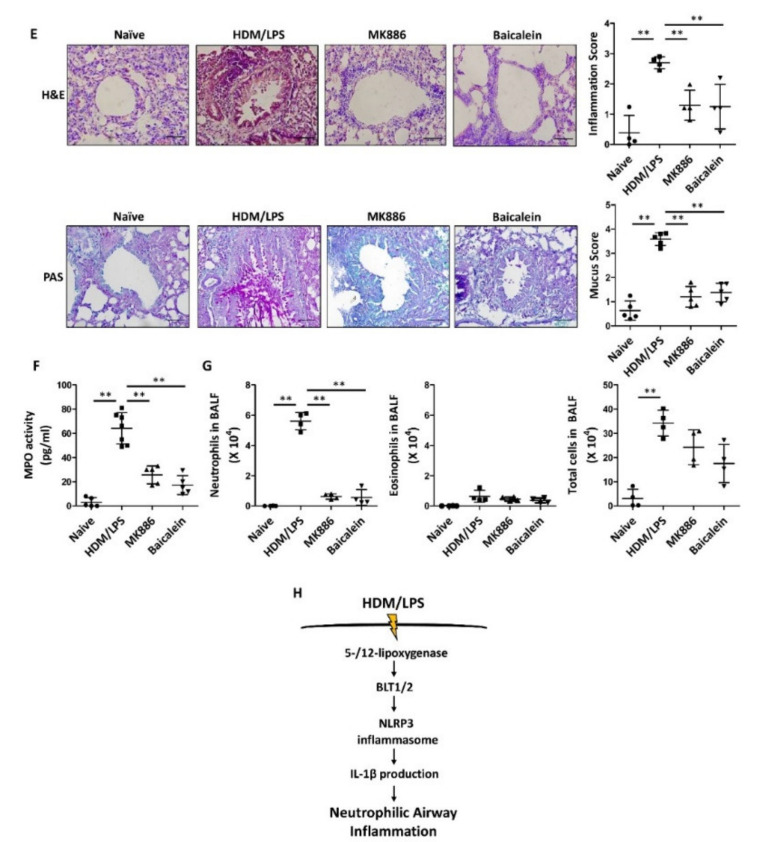
5/12-lipoxygenase are necessary for NLRP3 stimulation and IL-1β production in HDM/LPS-induced neutrophilic airway inflammation. The control DMSO, MK886 (5 mg/kg) and baicalein (75 mg/kg) were administered via i.p. injection 1 h before every challenge. Naïve designates control without any additions. (**A**) Levels of IL-1β, LTB_4_ and 12(S)-HETE in lung lysates were analyzed using ELISA. Data are shown as the mean ± SD (*n* = 5–7 per group). (**B**,**C**) The mouse lungs were homogenized, protein was isolated, and the levels of 5-/12-LOX, NLRP3, ASC and caspase-1 were assessed by Western blotting. Data are representative of three independent experiments with similar results. Quantification of NLRP3 and caspase-1 was provided by image J. (**D**) Immunofluorescence staining for NLRP3 (red, CY^TM^3) in lung tissues. Nuclei were stained with DAPI (blue) (400×). The images are representative of three independent experiments with similar results. (**E**) The lungs were excised, fixed, and stained with H&E and PAS. Peribronchial and perivascular lung inflammation were measured and scored (400×). Data are shown as the mean ± SD (*n* = 4 per group). (**F**) Levels of MPO in lung lysates were analyzed using ELISA. Data are shown as the mean ± SD (*n* = 5–7 per group). (**G**) Neutrophils, eosinophils, and total immune cells in BALF were obtained using Cytospin and stained with Diff-Quik. Data are shown as the mean ± SD (*n* = 4 per group). (**H**) Scheme of the ‘5/12-LOX-BLT1/2-NLRP3 inflammasome-IL-1β’ signaling cascade in HDM/LPS-induced neutrophilic airway inflammation. All experiments were performed in triplicate. * *p* < 0.05, ** *p* < 0.01 versus each control group.

Next, we determined whether the synthesis of BLT1/2 ligands by 5-/12-LOX was also associated with the stimulation of the NLRP3 inflammasome and IL-1β synthesis. To examine the roles of 5-/12-LOX, we used inhibitors of each enzyme. HDM-induced mice were administered the 5-LOX inhibitor MK886 (5 mg/kg) or the 12-LOX inhibitor baicalein (75 mg/kg) 1 h before each challenge by intraperitoneal injection. We found that the levels of IL-1β were significantly reduced by MK886 or baicalein (Figure 4A). Under these conditions, the levels of LTB_4_ and 12(*S*)-HETE were markedly suppressed by MK886 and baicalein, respectively (Figure 4A). Next, we observed that the expression levels of NLRP3, ASC complex and caspase-1 were markedly reduced by MK886 or baicalein (Figure 4B,C). By IF staining analysis, NLRP3 expression was also shown to be reduced by MK886 or baicalein (Figure 4D). Histological analysis demonstrated that MK886 or baicalein alleviated immune cell infiltration and mucus secretion in the airway compared to those of the control group (Figure 4E). Additionally, the administration of MK886 or baicalein significantly reduced the levels of MPO activity in lung lysates and the number of neutrophils in BALF (Figure 4F,G). Thus, these results suggest that the stimulation of the NLRP3 inflammasome and IL-1β synthesis are dependent on 5-/12-LOX, further supporting the roles of BLT1/2 in HDM/LPS-driven neutrophilic severe airway inflammation (summarized in Figure 4H).

## 4. Discussion

In the present study, we demonstrated that the LTB_4_ receptors BLT1 and BLT2 play an important role in the stimulation of the NLRP3 inflammasome and IL-1β synthesis in HDM/LPS-driven neutrophilic airway inflammation. In addition, we demonstrated that 5-/12-LOX functions upstream of BLT1/2 to mediate NLRP3 stimulation and IL-1β synthesis. Our findings may provide novel insight into the detailed mechanisms underlying the NLRP3 inflammasome and IL-1β synthesis observed in severe neutrophilic asthmatic airway inflammation.

The pathological phenotypes of asthma can be classified based on the inflammatory cell types that infiltrate the airway, such as eosinophilic, neutrophilic, or eosinophilic/neutrophilic mixed inflammation [17,18,19]. Mild to moderate asthma is typically Th2 lymphocyte associated, with eosinophilic airway inflammation, airway hyperresponsiveness and mucus hypersecretion [20,21,22,23]. The patients with these asthma subtypes respond well to corticosteroid therapies. However, moderate to severe types are more strongly associated with Th1/Th17-mediated responses and neutrophilic airway inflammation [24,25]. Recent studies have reported that high level of LPS, a component of the cell wall of Gram-negative bacteria, plays a role in aggravating allergen-induced asthma symptoms [26,27]. For example, neutrophils were dominantly recruited to airway exposed by high-dose LPS in allergen-induced asthma [28]. In accordance with these findings, bacterial infection has been associated with neutrophilic steroid-resistant severe asthma [17,29]. In addition, several groups have already reported that the addition of LPS during the HDM sensitization phase caused a neutrophil-dominant lung inflammation [30,31]. Additionally, we previously observed that the addition of LPS during the HDM sensitization phase induced neutrophil-dominant lung airway inflammation [6]. In that study, we demonstrated that BLT1/2 play critical roles in HDM/LPS-induced neutrophilic airway. However, it remains to be determined whether BLT1/2 are associated with NLRP3 stimulation and IL-1β synthesis in neutrophilic airway inflammation.

Recent studies have reported that the NLRP3 inflammasome plays an important role in directing Th1/Th17 responses in neutrophil-dominant severe asthma and several other inflammatory diseases that involve neutrophilic response [10,32,33,34,35,36,37]. NLRP3 is an intracellular sensor that detects a variety of exogenous or endogenous danger signals, resulting in the formation and activation of the NLRP3 inflammasome [38,39]. The multiprotein complex of the NLRP3 inflammasome consists of a sensor (NLRP3), an adaptor (apoptosis speck-like protein ASC; also known as PYCARD) and an effector (caspase 1). Thus, NLRP3 initiates inflammasome assembly by interacting with ASC, which recruits and activates pro-caspase-1 to generate active caspase-1 and then converts the cytokine precursor pro-IL-1β. Once activated, IL-1β is formed and released from immune cells. Subsequently, these proinflammatory cytokines trigger a series of inflammatory responses and pyroptotic cell death [40,41,42,43,44].

In particular, the proinflammatory cytokine IL-1β plays a critical role in neutrophil recruitment and activation, which contribute to the development of severe neutrophilic airway inflammation in the airways of the lungs [10,45,46]. In addition, elevated levels of IL-1β were found in the sputum of patients with neutrophil-dominant severe asthma [9]. Consistent with these reports, we found that IL-1β levels were significantly increased in HDM-induced neutrophilic airway inflammation (Figure 2A). Additionally, in accordance with previous results, the inhibition of IL-1β with an anti-IL-1β neutralizing antibody was shown to significantly reduce NLRP3 stimulation and neutrophil recruitment in HDM-induced mice (Figure 1 and Figure 2), suggesting that IL-1β plays a critical role in the pathogenesis of neutrophil-dominant airway inflammation [47,48,49].

In the present study, we found that the levels of BLT1 and BLT2, as well as the synthesis of their ligands LTB_4_ and 12(*S*)-HETE, were markedly upregulated in our neutrophil-dominant model and that blockade of BLT1/2 or 5-/12-LOX significantly reduced NLRP3 stimulation and IL-1β production, thus attenuating neutrophilic airway inflammation (Figure 3 and Figure 4). Consistent with the proposed role of BLT1/2 in neutrophilic inflammation [50,51,52]. Previous studies have reported that BLT1 is associated with neutrophil infiltration in several inflammatory diseases, including arthritis, spinal cord injury and allergic skin inflammation [50,51]. They reported that BLT1 blockade dramatically reduces neutrophil recruitment to inflammatory sites. In addition, BLT2 has proinflammatory activities upon cigarette smoke exposure, which enhances neutrophil adhesion and contributes to airway neutrophilia [52]. Furthermore, LTB_4_ and 12(*S*)-HETE were highly elevated in the sputum or serum of severe neutrophilic asthmatic condition [14,15]. Thus, along with the results of the present study, BLT1/2 likely contributes to the development of neutrophilic asthmatic airway inflammation.

## 5. Conclusions

In the present study, we found that the levels of BLT1 and BLT2 as well as the synthesis of their ligands LTB_4_ and 12(*S*)-HETE were markedly upregulated in our neutrophil-dominant model, and the blockade of BLT1 or BLT2 significantly reduced NLRP3 stimulation and IL-1β synthesis, thus attenuating neutrophilic airway inflammation (Figure 3 and Figure 4). Thus, our results suggest that the ‘5-/12-LOX-BLT1/2-NLRP3-IL-1β’ cascade significantly contributes to the development of neutrophil-dominant airway inflammation. This is the first report on the role of BLT1/2 in the regulation of NLRP3 stimulation and IL-1β synthesis in a neutrophilic airway inflammation mouse model, and our results may provide a perspective for the development of treatments for asthma patients with steroid resistance.

## Data Availability

Not applicable.
